# Supplementary material for: Evidence from COVID-19 Patients and Murine Studies for a Continuing Trend Towards Targeting of Nasopharyngeal Ciliated Epithelial Cells by SARS-CoV-2 Omicron Sublineages
Source: Viruses. 2025 Dec 17;17(12):1631. doi: 10.3390/v17121631 (PMC12737339; doi:10.3390/v17121631)
Supplement: Supplementary file 1 [file viruses-17-01631-s001.zip › Appendix A.pdf]

Participant Information Sheet and Consent Form

Cohorts I, II & III (enrol or continue)

Collection of Coronavirus COVID-19 outbreak samples in South Australia (COVID-19 SA)

**Short Title**	COVID-19 SA
**Protocol Number**	13050
**Principal Investigator**	Dr Anushia Ashokan<br>Dr Benjamin Reddi
**Location**	Royal Adelaide Hospital

This Participant Information and Consent Form is ten pages long. Please make sure you have all the pages of this document.

1. Introduction

You are invited to take part or continue your participation in this research project because you tested positive to the virus COVID-19.

This Participant Information Sheet/Consent Form tells you about the research project. It explains the tests involved. Knowing what is involved will help you decide if you want to take part in the research.

Please read this information carefully and take your time in making your decision. Ask questions about anything that you don't understand or want to know more about. Before deciding whether or not to take part, you may like to talk about it with a relative, friend or your usual doctor. Participation in this research is **voluntary**. If you don't wish to take part, you don't have to. If you decide to participate, you can stop at any time if you change your mind later on.

If you decide you want to take part in the research project, you will be asked to sign the consent section, or if in COVID-19 isolation you will be asked to provide verbal consent By signing or providing verbal consent you are telling us that you:

- Understand what you have read;
- Consent (agree) to take part in the research project;
- Consent (agree) to have the tests
- Consent (agree) to the use of your personal and health information as described.

You will be given a copy of this Participant Information and Consent Form to keep.

2. What is the purpose of this research?

The purpose of this research is to look at the immune response body creates to fight the COVID-19 infection. Information like this will help scientists and doctors to make vaccines and therapies. This will also allow us to understand the impact of vaccination on specific immune markers affected by COVID-19. We will also explore the relationship between immune response and symptoms of long COVID.

3. What does participation in this research involve?

Consent

Due to the nature of your COVID19 infection you may move between the different groups described in this study. If you are in ICU or a hospital ward and are able, you will be asked to provide either written or verbal consent depending on whether you are under COVID-19 isolation requirements.

If you are approached for this study as an outpatient you will be asked to provide written consent by signing and dating the form at the end of this information sheet.

Procedures and Tests

If you agree to take part, at each study visit blood samples (maximum 20mls in ICU, maximum 40ml on the ward & maximum 70 mls as an outpatient) will be collected, along with nasopharangeal swabs if you are in ICU, to look at the white blood cells and antibodies your body has created in response to infection with COVID-19. We will also investigate what genes are expressed by your immune system during your COVID-19 infection and in the period after you have recovered. We will also look at the COVID-19 virus to determine the level of infection and study the virus.

Study blood samples are sent in deidentified form to be processed at the University of Adelaide investigator laboratories. Research samples will be collected from study participants during their hospital stay or within an outpatient setting depending on where they are in the COVID-19 disease process.

There will be no change to your standard of care and treatment in regards to COVID-19 infection, whether you chose to participate in the study or not.

Due to the nature of COVID19 infection you may move between these groups while in hospital.

ICU Patients

Nasopharyngeal swabs may be/ have been taken on days 0, 5, 7, 10, 14 and weeks 4 & 12 whilst you remain in hospital.

Bloods may be/ have been collected on days 0, 2, 5, 7, 10, 14 and weeks 4, 12,24,48,72,96,120 & 144 whilst you remain in hospital, 18. (maximum 20ml per sample during ICU stay, and maximum 40ml during ward admission)

Depending on feasibility, samples may not be collected at every timepoint listed above.

Ward Patients

You may have (had) bloods collected on days 0, 5 &14 and weeks 4, 12, 24,48,72,96,120 & 144 whilst you remain in hospital (maximum 40ml per sample)

Outpatients

You may be asked to come back to the RAH Infectious Diseases outpatient clinic for study visits to have blood collected on weeks 4, 8, 12, 16, 24, 48, 72, 96, 120, & 144. (maximum 70ml per sample)

Costs

You will not receive payment for participating in this research project.

4. What do I have to do?

It is important that you follow the instructions provided by the study staff at all times. If you have questions, please speak with a member of the study team.

Your Responsibilities

- Appointments and Tests: it is important that you attend clinic appointments and tests that the research project requires. Please talk to a member of the study team if you have any concerns. Blood collections will be arranged to occur at the Royal Adelaide Hospital. Clinical Rooms.
- You will receive appointment notifications advising you of follow up study visits.

5. Other relevant information about the research project

In response to the current COVID-19 pandemic, collecting information about how our body's immune system 'remembers' this infection is crucial. This project will give us information on how our body fights the virus, which cells are integral in protection against infection and how long does the immunity last for. We will also study the virus to learn more about preventing future infections. This information is crucial for global public health to prevent future outbreaks of this virus and how to design the vaccines to protect us from infection in the future.

6. Do I have to take part in this research project?

Participation in any research project (including this one) is voluntary. If you do not wish to take part, you do not have to. If you decide to take part and later change your mind, you are free to withdraw from the research project at any time.

Your decision to take part or not to take part, or to take part and then withdraw, will not affect your routine care, your relationship with those treating you or your relationship with the Royal Adelaide Hospital.

If you decide to take part and then withdraw you are encouraged to contact the study staff who will explain the best way for you to discontinue your participation. We recommend you go through the study withdrawal processes that the study staff considers necessary. No further study visits will occur but if you agree, site staff may still check on your health by looking at your medical records, contacting your general practitioner or checking publicly available registries. Alternatively, you may withdraw and request that no further information be collected about you.

7. What are the alternatives to participation?

You do not have to take part in this research project to receive treatment at The Royal Adelaide Hospital. You will be made aware of any new information during the course of the study that may affect your willingness to participate.

8. What are the possible benefits of taking part?

This research project aims to increase global medical knowledge about COVID-19 and may help prevent future infections and help the design and development of COVID-19 vaccines; however, it may or may not benefit you directly.

9. What are the possible risks and disadvantages of taking part?

Blood collection will be performed by direct puncture of the vein. Drawing blood can cause localised pain, bruising or infection where the needle is inserted. Some people experience dizziness, fainting or an upset stomach when their blood is drawn

Nasopharyngeal swabbing of the back of the throat and then the inside of the nose can cause some local discomfort and trigger coughing. Rarely, swabbing of the inside of the nose may cause a small nosebleed

Upset/Distress

If you become upset or distressed from taking part in the study, the study staff will be able to arrange counselling or other appropriate support for you. Any counselling or support will be provided by qualified staff who are not members of the study team.

10. What will happen to my test samples?

Research Samples

Your research samples will be stored as de-identified samples. This means that your samples will be identified only by a code; they can be re-identified as yours by the Royal Adelaide Hospital study team but the laboratory based people working with the samples will not know your identity.

The research samples will be stored and studied at the University of Adelaide research laboratories and QIMR Berghofer Medical research Institute.

Your name or any other information that could directly identify you will not be stored with the samples or data. Your confidentiality will be protected at all times.

You should be aware that knowledge acquired through medical research conducted on your tissue sample may lead to discoveries that are of commercial value to the researcher and their institutions; however there will be no financial benefit to you or your family.

If your sample is needed for your clinical care, you or your doctor can ask to retrieve it from where it is stored. However, return may not be possible if there is no material left at the time of the request.

Genetic Testing

Your blood samples contain DNA and RNA, which makes up the genes that serve as the "instruction book" for the cells in our bodies. By using DNA and RNA from your blood samples, researchers will study part or all of your body's genetic sequence, known as your genome. The genome sequence will be read and this information will be stored.

Your genomic data will be used to find differences and similarities among people who tested positive to COVID-19. Your genomic data and health information will be studied along with information from other participants in this research project, and it will be stored for follow up studies. This is not a genetic risk study, and any sequence information collected will not be used for genetic risk predictions, or for diagnosis of other conditions.

Results will remain in the research database and are not planned to be returned directly to study participants or placed in participants' medical records. Genetic data from your samples may also be shared in public databases. Only de-identified data (stripped of all identifying information like name, address, etc.) is shared in public databases. Sharing genetic data can help researchers use research results to develop knowledge, products and procedures that can improve human health.

Withdrawal of Required Samples

If you no longer want your samples to be used in this research, you should tell study personnel. Some of the samples may have already been used, in whole or in part, as part of the research project. If tests have already been done on your sample(s) it will not be possible to withdraw those results. However, no further research will be done.

11. What if new information arises during this research project?

Medical research is a very difficult and time consuming process and by the time researchers have identified any important findings and these are executed in clinical medicine, the results will generally have no bearing on the participants that donate the samples. You may contact us to find out what projects your donated samples were used for.

Can I have other treatments during this research project?

Government of South Australia SA Health | Health Central Adelaide Local Health Network | THE UNIVERSITY of ADELAIDE

Yes, you should have all your normal treatments and care throughout this research project. Please keep the study personnel informed of your medications and treatments.

12. What if I withdraw from this research project?

If you wish to withdraw from this research project, please tell a member of the study team.

If you do withdraw your consent during the research project, study personnel will not collect additional personal information from you, although personal information already collected will be kept, to ensure the results of the research project can be measured properly and to comply with law. You should be aware that data collected up to the time you withdraw will form part of the research project results. You will be unable to remove the data that has already been collected.

13. Could this research project be stopped unexpectedly?

This research project may be stopped unexpectedly for a variety of reasons. These include:

- Decisions made by the researchers upon review of the data;
- Decisions made by local regulatory/health authorities.

14. What happens when the research project ends?

A member of the study team will inform you if continued participation in the research project is no longer in your best interest.

The results of this research project will be published in scientific medical journals and presented at conferences and other professional forums. Usually a number of years pass before the results of this type of research project are available. You can ask a member of the study team about this.

15. What will happen to information about me?

By signing the consent form you agree to the study team collecting and using personal information about you for the research project. All information collected for this research project will remain confidential. Your information will only be used for the purpose of this research project and it will only be used with your permission, except as required by law.

Information about you may be obtained from your health records held at this and other health services for the purpose of this research. By signing the consent form you agree to the study team accessing health records if they are relevant to your participation in this study. Information about your participation in this study will be recorded in your health records. Once the study has been completed, the the records will be retained in a locked storage facility for 15 years. The de-identified data from your participation in the study and test results will be stored by the Australian coordinating centre, for at least 20 years and may be retained for an indefinite period.

All your samples and data will be stored securely and only authorised study personnel including monitors, auditors, ethics committees and inspectors will have access to them. Your information will only be disclosed with your permission, except as required by law.

Neither your research project data nor your samples will be identified with your name. Instead, a participant code will be used by your study team in all documentation submitted for the research project. If the Sponsor transfers research project data to other researchers, appropriate protection will continue to be applied to such research project data. Only the coded data will be transferred. When the results of the research project are published in medical literature, your identity will remain confidential.

In accordance with relevant Australian and/or South Australian privacy and other relevant laws, you have the right to request access to the information collected and stored by the study team about you. You also have the right to request that any information with which you disagree be corrected. Please contact the study team member named at the end of this document if you would like to access your information.

16. Complaints and compensation

This study has been approved by the The Central Adelaide Health Local Network Human Research Ethics Committee and Research Governance Office. Any person with concerns or complaints about the conduct of this study should contact the Research Office who is nominated to receive complaints from research participants on 08 7117 2209 .

If you suffer any injuries or complications as a result of this study, you should contact the study staff as soon as possible and you will be assisted in arranging appropriate medical treatment.

In the event of loss or injury, the parties involved in this research project have agreed that you may have a right to take legal action to obtain compensation for any injuries or complications resulting from the study. Compensation may be available if your injury or complication is sufficiently serious and is caused by unsafe equipment, or by the negligence of one of the parties involved in the study (for example the researcher or the hospital). If you receive compensation that includes an amount for medical expenses, you will be required to pay for your medical treatment from those compensation monies. You do not give up any legal rights to compensation

by participating in this study. If you are not eligible for compensation for your injury or complication under the law, but are eligible for Medicare, then you can receive any medical treatment required for your injury or complication free of charge as a public patient in any Australian public hospital.

17. Who is organising and funding the research?

This research is being led by the University of Adelaide in collaboration with Central Adelaide Local Health Network. The project is funded by grant funding from The Hospital Research Foundation and the Health Services Charitable Gift Board.

No member of the research team will obtain any personal financial benefit from their involvement in this research project.

18. Who has reviewed the research project?

All research in Australia involving humans is reviewed by an independent group of people, called a Human Research Ethics Committee (HREC). This research project has been reviewed and given approval by CALHN : 13050.

This research project will be carried out according to the *National Statement on Ethical Conduct in Human Research (2007 and updates)* produced by the National Health and Medical Research Council of Australia. This statement has been developed to protect the interests of people who agree to participate in human research studies.

19. Further information and who to contact

The person you may need to contact will depend on the nature of your query.

If, at any time, you would like any further information about this research project or if you have any medical problems which may be related to your involvement in the research project , you can contact the Principal Study Doctors Dr Anushia Ashokan on 08 7074 0000 or Dr Benjamin Reddi on 7074 1781 or any of the following people:

Clinical contact persons

Name	Ms Catherine Ferguson
Position	Study Coordinator
Telephone	08 7074 2788
Email	Health.InfectiousDiseasesResearch@sa.gov.au

Name	Sarah Doherty
Position	Nurse Unit Manager – ICU Research
Telephone	08 7074 1801
Email	Sarah.Doherty@sa.gov.au

Complaints about this research

Should you have concerns about your rights as a participant in this research, or you have a complaint about the manner in which the research is conducted, it may be given to the researcher, or, if an independent person is preferred, to:

Name	Ms Bernadette Swart
Position	Manager, CALHN Research Office
Address	RAH, Level 3, 3D460.02, Port Road, Adelaide 5000
Telephone	(08) 7117 2209
Email	Health.CALHNResearchGovernance@sa.gov.au

Reviewing HREC approving this research and HREC Executive Officer Details

If you have any complaints about any aspect of the project, the way it is being conducted or any questions about being a research participant in general, then you may contact:

Reviewing HREC name	Central Adelaide Health Local Network HREC
HREC Executive Officer	Mr Ian Tindall
Telephone	08 7117 2229
Email	Health.CALHNResearchEthics@sa.gov.au

<u>Verbal</u> Participant Consent Form

Cohorts I, II & III

Collection of Coronavirus COVID-19 outbreak samples in South Australia (COVID-19 SA)

**Short Title**	COVID-19 SA
**Protocol Number**	13050
**Principal Investigator**	Dr Anushia Ashokan<br>Dr Benjamin Reddi
**Location**	Royal Adelaide Hospital

<u>Verbal Declaration by Participant</u>

1. I have read the Participant Information Sheet or someone has read it to me in a language that I understand.
2. I understand the purposes, procedures and risks of the research described in the project.
3. I have discussed my participation in this research project with the member of the study team named below. I have had the opportunity to ask questions and I am satisfied with the answers I have received.
4. I consent to my doctors, other health professionals, hospitals or laboratories outside this institution releasing information concerning my condition and treatment which is needed for this research project and understand that such information will remain confidential
5. I freely consent to participate in the research project as described and understand that I am free to withdraw at any time during the study without affecting my future health care
6. I consent to the storage and use of samples taken from me, as described in the relevant section of the Participant Information Sheet, for:

   This specific research project ☐ Yes ☐ No

   Other research that is closely related to this research project ☐ Yes ☐ No

   Any future research ☐ Yes ☐ No
7. I consent for the use of my collected information in future research ☐ Yes ☐ No
8. I agree to my medical records (paper and electronic) being accessed for both the duration and follow up of the study; COVID-19 SA ☐ Yes ☐ No
9. I understand that I will be given a signed copy of this document to keep.

10. I understand that I can withdraw my consent to participate in the research project by filling in a “Withdrawal of Consent” form, or by telling a study team member.

Name of participant (please print) ____

Name of Interpreter if used (please print)	____________________
Signature ____________________	Date ____________________

Declaration by Principal Investigator or Senior Researcher

I have given a verbal explanation of the research project, its procedures and risks and I believe that the participant has understood that explanation.

Principal Investigator/<br>Senior Researcher† (please print)	____________________
Signature ____________________	Date ____________________

†A senior member of the research team must provide the explanation of, and information concerning, the research project.

Note: All parties signing the consent section must date their own signature.

A copy of this consent will be sent/emailed to the participant

<u>Written</u> Participant Consent Form

Cohorts I, II & III

Collection of Coronavirus COVID-19 outbreak samples in South Australia (COVID-19 SA)

**Short Title**	COVID-19 SA
**Protocol Number**	13050
**Principal Investigator**	Dr Anushia Ashokan<br>Dr Benjamin Reddi
**Location**	Royal Adelaide Hospital

Declaration by Participant

1. I have read the Participant Information Sheet or someone has read it to me in a language that I understand.
2. I understand the purposes, procedures and risks of the research described in the project.
3. I have discussed my participation in this research project with the member of the study team named below. I have had the opportunity to ask questions and I am satisfied with the answers I have received.
4. I consent to my doctors, other health professionals, hospitals or laboratories outside this institution releasing information concerning my condition and treatment which is needed for this research project and understand that such information will remain confidential
5. I freely consent to participate in the research project as described and understand that I am free to withdraw at any time during the study without affecting my future health care
6. I consent to the storage and use of samples taken from me, as described in the relevant section of the Participant Information Sheet, for:

   This specific research project ☐ Yes ☐ No

   Other research that is closely related to this research project ☐ Yes ☐ No

   Any future research ☐ Yes ☐ No
7. I consent for the use of my collected information in future research

   ☐ Yes ☐ No
8. I agree to my medical records (paper and electronic) being accessed for both the duration and follow up of the study; COVID-19 SA ☐ Yes ☐ No
9. I understand that I will be given a signed copy of this document to keep.

10. I understand that I can withdraw my consent to participate in the research project by filling in a “Withdrawal of Consent” form, or by telling a study team member.

Name of Participant (please print)	________________________
Signature ________________________	Date ________________________

Name of Interpreter if used (please print)	________________________
Signature ________________________	Date ________________________

Declaration by Principal Investigator or Senior Researcher

I have given a verbal explanation of the research project, its procedures and risks and I believe that the participant has understood that explanation.

Principal Investigator/<br>Senior Researcher† (please print)	________________________
Signature ________________________	Date ________________________

†A senior member of the research team must provide the explanation of, and information concerning, the research project.

Note: All parties signing the consent section must date their own signature.

A copy of this consent will be sent/emailed to the participant

Form for Withdrawal of Participation

Cohorts I, II & III

Collection of Coronavirus COVID-19 outbreak samples in South Australia (COVID-19 SA)

**Short Title**	COVID-19 SA
**Protocol Number**	13050
**Principal Investigator**	Dr Anushia Ashokan<br>Dr Benjamin Reddi
**Location**	Royal Adelaide Hospital

Declaration by Participant

I wish to withdraw from participation in the above research project and understand that such withdrawal will not affect my routine treatment, my relationship with those treating me or my relationship with the Royal Adelaide Hospital.

Name of Participant (please print) ______________________________

Signature ______________________________ Date ______________________________

In the event that the participant's decision to withdraw is communicated verbally, the Study Doctor/Senior Researcher will need to provide a description of the circumstances below.

Declaration by Study Doctor/Senior Researcher†

I have given a verbal explanation of the implications of withdrawal from the research project and I believe that the participant has understood that explanation.

Name of Study Doctor/ Senior Researcher† (please print)	______________________
Signature ______________________	Date ______________________

† A senior member of the research team must provide the explanation of and information concerning withdrawal from the research project.

Note: All parties signing the consent section must date their own signature.
