# Supplementary material for: Evidence from COVID-19 Patients and Murine Studies for a Continuing Trend Towards Targeting of Nasopharyngeal Ciliated Epithelial Cells by SARS-CoV-2 Omicron Sublineages
Source: Viruses. 2025 Dec 17;17(12):1631. doi: 10.3390/v17121631 (PMC12737339; doi:10.3390/v17121631)
Supplement: Supplementary file 1 [file viruses-17-01631-s001.zip › Figures S1&S2.pdf]

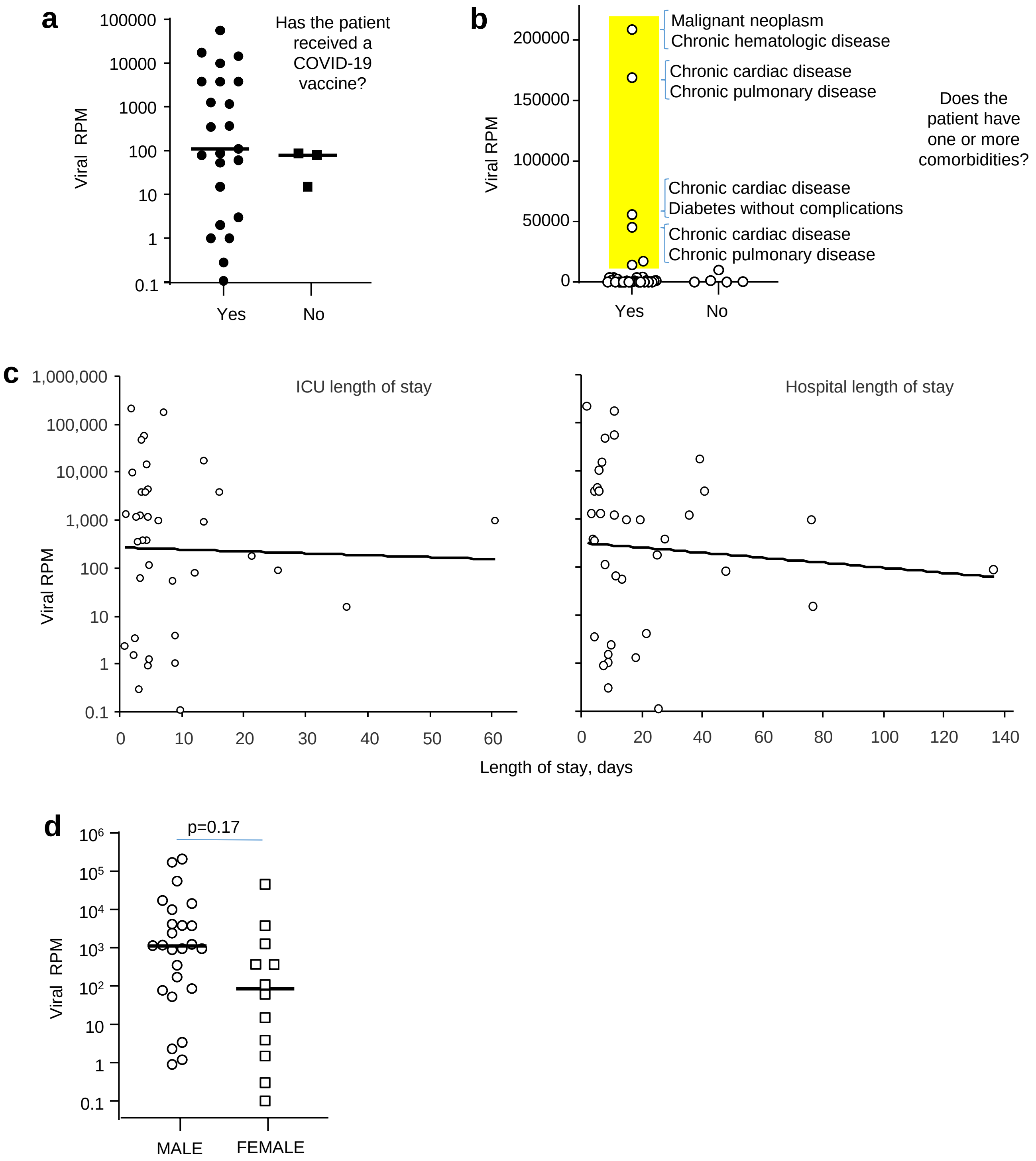

Figure S1. Non-significant correlations with viral load. (**a**) COVID-19 vaccination status and the relationship to viral RPM. There was no significant difference between groups; however, with only 3 patients in the No group, the power of this analysis is marginal. (Vaccination status was not known for a number of patients). (**b**) Higher viral RPM were associated with patients that had one or more comorbidities (yellow shading); however, this did not reach significance. The comorbidities for four patients with the highest viral RPM are briefly described. Note there were only 5 patients with no comorbidities. (**c**) Length of stay in ICU or hospital plotted against viral RPM. No significant correlations emerged. (**d**) Viral RPM was not significant between males and females. Statistics by Kolmogorov-Smirnov test (unequal variance).

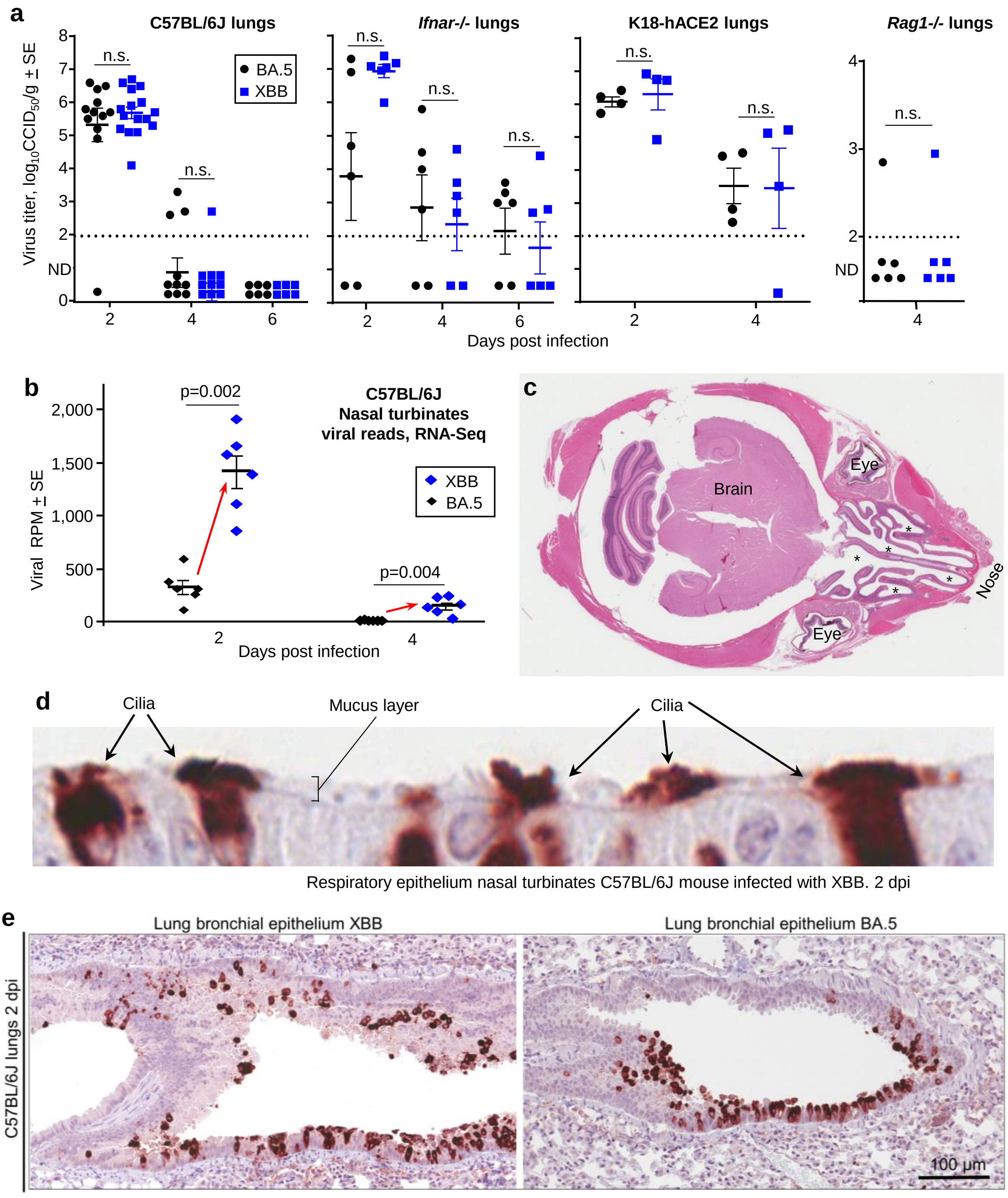

Figure S2. (**a**) Corresponding XBB and BA.5 lung virus titres for the same individual mice for which nasal turbinate titres are shown in Fig. 4b. Labelling as in Fig. 4b. (**b**) The C57BL/6J experiment shown in Fig. 4b was repeated, with viral RPM in nasal turbinates determined by RNA-Seq as described (ref 28) (and as undertaken for the COVID-19 patient nasopharyngeal swabs described in Fig. 1a). Labelling as in Fig. 4b. (**c**) A whole head section of a C57BL/6J mouse stained with H&E to illustrate orientation of sections used for IHC. * sinus spaces of the nasal turbinates. (**d**) Enlargement of the IHC image showing cilia staining in Fig. 5b. (**e**) IHC of C57BL/6J lung from XBB and BA.5 infected mice; similar levels of staining for both viruses were seen in bronchial epithelial cells, consistent with viral titer data in Fig S2a. Infection of alveoli was rare (not shown).
